# Visually Activating Pathogen Disgust: A New Instrument for Studying the Behavioral Immune System

**DOI:** 10.3389/fpsyg.2018.01397

**Published:** 2018-08-08

**Authors:** Paxton D. Culpepper, Jan Havlíček, Juan David Leongómez, S. Craig Roberts

**Affiliations:** ^1^Division of Psychology, University of Stirling, Stirling, United Kingdom; ^2^Department of Zoology, Charles University, Prague, Czechia; ^3^Faculty of Psychology, El Bosque University, Bogotá, Colombia

**Keywords:** disgust sensitivity, disgust images, visual stimuli, disgust prime, disgust scale

## Abstract

The emotion of disgust plays a key role in the behavioral immune system, a set of disease-avoidance processes constituting a frontline defense against pathogenic threats. In the context of growing research interest in disgust, as well as recognition of its role in several psychiatric disorders, there is need for an improved understanding of behavioral triggers of disgust and for adequate techniques to both induce disgust in experimental settings and to measure individual variability in disgust sensitivity. In this study, we sought to address these issues using a multi-stage, bottom-up approach that aimed first to determine the most widespread and effective elicitors of disgust across several cultures. Based on exploratory factor analysis of these triggers, revealing four main components of pathogen-related disgust, we then generated a novel visual stimulus set of 20 images depicting scenes of highly salient pathogen risk, along with paired control images that are visually comparable but lack the disgust trigger. We present a series of validation analyses comparing our new stimulus set (the Culpepper Disgust Image Set, C-DIS) with the most commonly used pre-existing set, a series of 7 images devised by Curtis et al. (2004). Disgust scores from participants who rated the two image sets were positively correlated, indicating cross-test concordance, but results also showed that our pathogen-salient images elicited higher levels of disgust and our control images elicited lower levels of disgust. These findings suggest that the novel image set is a useful and effective tool for use in future research, both in terms of priming disgust and for measuring individual differences in disgust sensitivity.

## Introduction

The emotion disgust is commonly characterized as a negatively valenced affective state consisting of a set of interlinked cognitive, behavioral, and physiological processes (Rozin et al., 1994). It has been proposed that these processes represent a putative adaptation to avoid disease, principally functioning to minimize direct contact with threats of infectious microorganisms, i.e, pathogens (Curtis et al., 2004; Oaten et al., 2009). Earlier literature suggests the role of disgust to be primarily concerned with avoiding oral ingestion of noxious stimuli (e.g., Rozin et al., 2000), but, based on the understanding that bacterial and viral infections can be transmitted through bodily excretions and secretions, Curtis and colleagues extended this idea to describe it as an adaptation that evolved to “…prevent the acquisition of infectious diseases” in general (Curtis et al., 2004, p.132), rather than simply via oral ingestion. While research suggests that disgust may also cross into sexual and moral domains (see Tybur et al., 2009), the pathogen disgust domain is likely the adaptation's foundation.

More recently, disgust has been cast as a key component in the concept of the behavioral immune system (BIS), an evolved set of disease-avoidance processes which serves as a psychological first line of defense against pathogen threats in the environment (Schaller, 2006; Lieberman and Patrick, 2014). The BIS is defined as behaviorally analogous to the classic immune system, consisting of a collaborative suite of evolved psychological mechanisms responsible for (1) processing and inferring potential risks of infection through perceptual cues, (2) activating aversive emotional and cognitive responses, which (3) motivate avoidance behaviors in order to neutralize the perceived threat (Schaller, 2006, 2011; Fincher and Thornhill, 2012). The similarities and overlap between pathogen disgust and the BIS are overtly apparent. In fact, researchers argue that they are functionally the same, declaring the distinction as no longer necessary or useful (Lieberman and Patrick, 2014). Whether this is the case or not, the initial step in activating the BIS is to prime the corresponding processing and inferential mechanisms with perceptual cues that “trigger” pathogen disgust.

### Visual cues to disgust

Several studies have demonstrated that experimentally priming people with pathogen-relevant cues can activate the BIS and alter their subsequent behavior (Tybur et al., 2014). Such primes can be introduced through different sensory modalities, including olfactory, tactile, and visual cues (Tybur et al., 2014). For example, after experimental exposure to odor evocative of feces, participants reported increased intention to use condoms compared to participants in a control condition (Tybur et al., 2011).

However, most studies conducted to date have employed the use of visual cues to pathogens (e.g., Faulkner et al., 2004, studies 5 and 6; Wu and Chang, 2012, studies 2 and 3), but these often have methodological or experimental limitations. For example, Faulkner and colleagues exposed participants to an 11-picture “Disease slide show,” noted as appropriate for teaching health education, that depicted “various ways that diseases are transmitted in daily life” (2004, p.345).There were some limitations to these images: one showed a woman in a kitchen attempting to kill cartoon germs, while another depicted a microscopic view of a hair with bacteria surrounding it, with the label “Hair Bacteria.” The process by which these images were chosen or validated as effective BIS triggers was not explained, and the use of descriptive text labels arguably defeats the purpose of visually cueing the BIS. Furthermore, the control condition consisted not of images that were similar but lacking in disease relevance, but was rather an “Accidents slide show” showing a series of potential safety threats (e.g., “School Bus Hazards,” “Electricity and Water Don't mix”). In another study (Wu and Chang, 2012), participants were exposed to a 10-image slide show depicting maggots and gory wounds, which is arguably more ecologically valid than those used by Faulkner et al., but the process of image selection and validation was also not described (and they similarly employed an “accident” slide show as the control condition). Moreover, neither of these studies asked their participants to rate the images for disgust, which would have provided evidence as to the effectiveness of the images in eliciting disgust. Several other image sets have been devised and validated to study affective responses generally, including disgust, such as the International Affective Picture System (IAPS: Lang et al., 2008), the Nencki Affective Picture System (NAPS: Marchewka et al., 2014), the Geneva Affective Picture Database (GAPED: Dan-Glauser and Scherer, 2011), and the Emotional Picture System (EmoPicS: Wessa et al., 2010); however, none of these were specifically designed as instruments to be used in the study of disgust.

For over a decade, the main set of photo stimuli produced specifically for the purposes of studying disgust was the set by Curtis et al. (2004). Devised from an evolutionary perspective, this set depicts 7 images of disease-salient stimuli (bowl of bodily fluid, feverish face, a crowded train carriage, red-green secretion on a towel, open wound, intestinal parasites, a louse) and a control set of 7 images that contextually matched each individual disease photo but lacked its corresponding disease relevance. Participants from across the world rated the disease-salient photos as more disgusting than their disease-free counterpart, providing support for the tested hypothesis that disgust evolved to motivate pathogen-avoidance (Curtis et al., 2004), and exposure to these disease-salient images has been shown to influence behavior (e.g., strategic mate preferences, Little et al., 2011; but see McIntosh et al., 2017). Despite these advantages, the image set is relatively small and the range of disgust elicitors is thus limited. Furthermore, although they demonstrably elicit the emotion of disgust, it seems likely that there are other kinds of stimuli that would elicit for more disgust; for example, there is no representation of fecal stimuli that appears to be one of the most evocative triggers of disgust around the world (e.g., Curtis and Biran, 2001).

More recently, as the current study neared completion, Haberkamp et al. (2017) developed their own validated set of images: the DIsgust-RelaTed-Images (DIRTI) picture set. The DIRTI was designed from a clinical perspective through a top-down approach, targeting six preselected disgust categories considered to play a role in psychiatric disorders: food, animals, body products, injuries/infections, death, and hygiene. It consists of 300 images, each category containing 40 related disgust images and 10 matched neutral images, and importantly these are copyright-free and accessible for re-use. We therefore think this set is extremely useful; however, one potential objection is that the categories were selected in top-down fashion by the researchers (similar to the image selection by Curtis et al.), rather than being driven by a bottom-up quantitative approach to category and item selection and with cross-cultural input.

### The present study

Against this background, we set out to develop a cross-culturally validated set of reliable visually priming stimuli for use in the study of disgust. To do this, we employed a multi-stage, bottom-up item-generation process modeled after methods used to generate other widely used instruments, such as the Three Domain Disgust Scale (Tybur et al., 2009), the original Disgust Scale (Haidt et al., 1994) and the Liverpool and Singaporean odor perception scales (Ferdenzi et al., 2011), and followed guidance on scale construction from Spector (1992). Each stage was necessary to increase the chances of generating images built from the most comprehensive list of possible universal disgust triggers.

In Stage 1 we asked a large cross-cultural sample of people about the five most disgusting items that came to mind. The intent was to assemble the widest possible range of items that individuals consider to be disgusting. We then filtered the item set (e.g., removing duplicates) while retaining the range, scope, and novelty of the original set (Stage 2) and had an independent set of raters score these items for disgust, providing a hierarchical ranking of the retained items and revealing those which were most commonly and consistently associated with disgust (Stage 3). We then extracted items that were determined to fall within the pathogen domain of disgust (Stage 4), used factor analysis to understand underlying structure of the remaining items (Stage 5), and adopted a set of decision rules to guide the selection and generation of 20 image-items and their controls (Stage 6; the final set of 20 paired images are hereafter referred to as the Culpepper Disgust Image Set, C-DIS). Finally, in Stage 7 we collected ratings of disgust elicited by these new disgust and control images and compared these responses with those obtained for the most commonly used images in previous disgust research (those by Curtis et al., 2004).

We developed the C-DIS with two potential uses in mind. First, exposure to the pathogen-salient image set could be used to more strongly elicit disgust in experiments where this may be the desired goal. For example, in studies in which experimenters wish to induce immunological activation, it would be useful to have the most disgusting set available. Second, it could provide a useful tool to assess individual participants' pathogen disgust sensitivity, for example by comparing the mean disgust scores that participants attach to the pathogen-salient and pathogen-free images.

We reasoned that, to be considered an improvement over the Curtis et al. image set, the C-DIS must meet specific criteria: it must elicit (1) a significantly larger overall mean disgust score for the pathogen-salient images compared to the pathogen-salient images in the Curtis set, (2) no significant increase (or some reduction) in the overall mean disgust score for the pathogen-free images compared to the disgust score for the pathogen-free images in the Curtis set, and therefore (3) a significantly larger difference in disgust scores given to the pathogen-salient and pathogen-free images compared to that of the Curtis set. Meeting these criteria would provide evidence to suggest that the C-DIS will more effectively trigger pathogen disgust, thus enabling more reliable manipulation of disgust and the BIS in future studies and across cultures.

## Method

### Ethics statement

This study received ethical approval from the General University Ethics Panel at the University of Stirling and adhered to the ethical guidelines of the British Psychological Society and the American Psychological Association. All participants provided prior informed consent (via the online survey) in accordance with the Declaration of Helsinki. No reward was offered for participation in any stage.

### Stage 1: the disgust item survey

#### Survey distribution

An online survey was generated which asked participants their age and gender, and then asked them to freely and in no particular order list 5 items (i.e., objects, scenarios, etc.) that they considered to be the most disgusting that came to mind. The survey was translated from English into two other languages (Czech, Spanish) by two bilingual researchers. The link to the English version was distributed across social media (e.g., Facebook, Twitter), which included mostly individuals from North America, the UK, and other English-speakers from other parts of the world, as well as to psychology students and staff at the University of Stirling in Scotland. The link for the Czech version was distributed to participants using a Facebook-based snowball method (Flegr and Kuba, 2016). The link to the Spanish version was distributed to staff and students at El Bosque University and the University of La Sabana in Colombia, several of whom also posted it on social media.

#### Participants

The surveys collectively garnered 865 total respondents: English version (*N* = 212), Czech version (*N* = 434), Spanish version (*N* = 219). The responses from the Spanish and English version surveys were filtered by removing all respondents that listed <3 of the requested 5 disgust items (Spanish: *N* = 179; English: *N* = 134 participants retained). Due to the larger number of Czech respondents, the translator selected only the respondents that listed all of the 5 disgust items, leaving 225 cases. She then removed every third respondent and translated the remaining 150 cases. Three of those were under age 18 and therefore removed (*N* = 147). To check for participants who responded to the survey more than once we assessed the IP addresses for duplicates. One duplicate IP address was discovered in the Colombian data, however, this is likely because the responders were students or staff at the same university. This resulted in a final total of 460 participants, including 114 men (24.8%), 344 women (74.8%), and 2 transgender (0.4%), with an overall mean age 31.84 ± *SD* 12.22 (range 18–69). Each survey version was responded to by individuals from a range of different global regions. More detailed descriptive statistics of participants for each individual survey version and the list of the countries are provided in ESM 1 and ESM2, respectively.

### Stage 2: disgust item reduction

#### Decision rules for disgust item reduction

Responses from the Czech and Colombian surveys were translated into English by the same two bilingual speakers. Responses from all three surveys were collated, providing a total of 2,287 disgust item responses (see ESM 2). A set of decision rules was followed to facilitate item-reduction.

First we removed verbatim duplicate responses and responses that describe the same item through similar words, e.g., we assumed, for example, “cruelty to animals” and “the smell of fish” to be equivalent to “animal cruelty” and “fish smell,” respectively. Items were retained if they appeared to describe something conceptually or contextually different, e.g., we retained both “touching spiders” and “spiders.” The second rule served to generalize the responses where appropriate, e.g., “Czech politics” was altered to simply “politics.” A third rule served to remove responses that were either too specific or not specific enough. For example, “Minister of Finance” was removed as not all governments have this position and because it implies a specific person who holds that position in that specific participant's country/government. Other items referring to specific individuals such as “my ex-husband” were also removed.

A further step was performed to help make the responses more comprehensible in subsequent stages by including brief descriptions to clarify some items for raters who may not know the meaning of, or have experience with, the regional vernacular regarding some items. For example, “touching the holding tubes in the public transport” was changed to “touching the holding tubes (hand-rails, etc.) in the public transport” and “the smell of the bathrooms in tube” was amended to “the smell of the bathrooms in tube (underground train).” Finally, responses such as “none” were also removed.

### Stage 3: disgust item rating task

#### Task objectives

The remaining 773 disgust items were then each rated for levels of disgust. A separate group of 20 participants (10 men, mean age ± *SD* = 38.7 ± 8.3, range 23–47; 10 women, age 34.2 ± 12.9, range 19–53) from the UK were recruited via email for this task. Participants rated each individual item for disgust on an 11-point scale (0 = *not at all disgusting*, 10 = *extremely disgusting*). The item-ratings were then standardized to *z*-scores for each of the 773 items. There was high concordance among raters across these items (Cronbach's α = 0.925). Ratings were then summed across raters to provide a mean score for each item, which were then ranked in descending order. Of these ranked disgust items, only the items within the upper quartile of disgust ranking were retained (*N* = 193) for use in Stage 4 (see ESM 3 for the item list).

### Stage 4: item categorization

#### Task objectives

In this stage, the remaining 193 items were categorized into major disgust domains—“pathogen,” “sexual,” or “moral” (see Tybur et al., 2009); or as “other” if the item did not fall into one of Tybur et al.'s three domains. Three raters (2 men and 1 woman), each familiar with Tybur et al.'s domain categorization, indicated to which domain they would assign each individual item. A Cronbach's *alpha* reliability test performed on their ratings indicated high inter-rater reliability (for all 3 raters α = 0.934).

Since the aim of this study was to select items related to the pathogen domain, items were retained if at least one researcher rated the item as relating to pathogen risk. Other items that were unanimously rated as belonging to the “moral” (e.g., “cruelty to animals,” “abuse to spouse,” “senseless murder,” “racism”) or “other” (e.g., “the sound of breaking bones”) were removed. No items were unanimously rated as “sexual” domain items. Although several items were labeled as “sexual” by two raters (e.g., “incest,” “animal intercourse (bestiality/zoophilia)”), these were retained because the third rater categorized these in the pathogen risk category. This step resulted in 131 remaining pathogen items (listed in ESM 4).

Finally, it was then necessary to perform a further reduction and unification procedure on the remaining items as it would not be possible to effectively, ethically, or unambiguously represent some items in an image. For instance, due to the difficulty of effectively depicting scenarios that describe auditory and tactile stimuli, such items were removed, e.g., “the crunch it makes when biting into a cartilage or tendon,” “burping in someone's face,” “eating something alive and feeling its movement in my mouth.” Items which could not be accurately assessed in an image were removed (e.g., “sperm other than from my partner and especially from a homeless person,” “bad or unpleasant odors”). Several items were related to “unwashed genitals” which could not ethically be represented and were removed. Several more were extremely similar and were unified into one item (e.g., “human entrails” and “gutted human bodies” were combined into “human entrails”; similarly “cat vomit,” “children's vomit,” and “vomit” were combined into “vomit”). Following this, 64 items (shown in Tables 1, 2) were retained.

**Table 1:** The 64 pathogen disgust items listed in ranked order of disgust rating from Stage 5.

**Overall rating**	**Disgust items**
5.21	Ingesting fecal matter
4.9	Eating uncooked rotting masses (any)
4.82	Rotting flesh crawling with worms
4.66	Worms in the food (where don't belong)
4.54	Maggots in wound of a living human
4.37	Decomposing human carcass
4.31	Eating a cockroach
4.26	Parasites/worms that grow in humans
4.17	Flesh-eating disease (parasites/bacteria)
4.04	Gaping infected wounds oozing pus
3.97	Body parasites
3.92	Really dirty, fungus-infected toenails
3.89	Sewage
3.87	Decomposing animal carcass
3.87	Intestinal parasites
3.86	Dead, disfigured body
3.82	Vomit
3.81	Rotting meat
3.79	Kissing someone with disgusting lips
3.75	Dirty sanitary items
3.69	Dirty or unflushed toilets
3.68	Maggots
3.64	Bugs, flies in food
3.63	Exposed intestines
3.62	When people eat their snot/bogeys
3.58	Liquid that comes out of the rubbish
3.57	A dog eating feces
3.56	Human entrails
3.55	Human feces
3.54	Rotting garbage
3.54	Open animal carcass
3.48	Bad dental hygiene, black teeth, decay
3.48	Stepping in dog feces
3.47	A baby diaper/nappy full of diarrhea
3.46	Bloody phlegm
3.34	Animal entrails
3.33	Bad body odor
3.28	Phlegm on sidewalks
3.23	Halitosis (bad breath)
3.18	The smell of garbage
3.12	Mucus, phlegm, snot
3.07	Exposed brains
3.05	Crawling swarms of insects
3.04	Cockroaches
3	Skin infections/diseases
2.99	Ball of hair in communal showers
2.91	Putrid or stagnant water
2.90	A gob of spit in the street
2.89	Sour milk
2.84	The bad odor of feet
2.83	Hair in your food
2.78	When people chew with mouth open
2.77	Long and dirty finger nails
2.75	Eating animal organs - brain, liver, etc.
2.72	Sloppy eaters
2.72	Severe acne (whiteheads, pus, etc.)
2.68	Close-up of a mouth while eating
2.64	Moldy food
2.64	Dirty scalp
2.63	Fat slobs who look filthy
2.61	Severe injuries (fractures, wounds)
2.60	Dog shit
2.55	Open wounds
2.30	Tumors

**Table 2:** PCA factor loadings of the 64 pathogen items for the four-factor model.

**Disgust item**	**Four factors**			
	**Hygiene Issues**	**Parasite /Infection**	**Food/Environmental**	**Injury /Viscera**
**FACTOR LOADINGS > 0.512**				
Halitosis (bad breath)	**0.720**	0.228	0.237	0.119
Dirty or unflushed toilets (1)	**0.626**	0.266	0.311	0.169
Bad body odor	**0.736**	0.206	0.277	0.067
Close-up of a mouth while eating	**0.694**	0.003	0.089	0.085
Dirty sanitary items (1)	**0.634**	0.252	0.369	0.176
Human feces	**0.578**	0.182	0.320	0.268
Hair in your food	**0.598**	0.286	0.187	0.167
Ball of hair in communal showers (e.g., the dorms)	**0.671**	0.275	0.227	0.184
When people eat their snot/bogeys (boogers) (1)	**0.590**	0.280	0.311	0.276
Bloody phlegm	**0.544**	0.479	0.000	0.284
Mucus, phlegm, snot	**0.696**	0.350	0.182	0.205
Long and dirty finger nails	**0.640**	0.462	0.054	0.179
Fat slobs who look filthy	**0.541**	0.358	0.185	0.025
When people chew with their mouth open	**0.753**	−0.144	0.155	0.111
Sloppy eaters	**0.676**	−0.003	0.252	0.147
Dirty scalp	**0.601**	0.500	0.155	0.128
Bad dental hygiene, black teeth, tooth decay (1)	**0.654**	0.443	0.058	0.144
The bad odor of feet	**0.742**	0.247	0.247	0.117
A gob of spit in the street	**0.697**	0.045	0.342	0.087
Phlegm on sidewalks	**0.699**	0.101	0.283	0.216
Flesh-eating disease (parasites, bacteria) (2)	−0.023	**0.617**	0.169	0.316
Body parasites	−0.043	**0.654**	0.371	0.329
Eating a cockroach	0.268	**0.575**	0.227	0.213
Cockroaches	0.241	**0.554**	0.271	0.081
Parasites/worms that grow in humans	0.131	**0.797**	0.186	0.170
Intestinal parasites	0.131	**0.794**	0.139	0.180
Maggots	0.218	**0.576**	0.327	0.287
Maggots in the wound of a living human	0.123	**0.725**	0.268	0.289
Really dirty, fungus-infected toenails (2)	0.474	**0.652**	0.037	0.086
Worms in the food (where they don't belong) (2)	0.261	**0.573**	0.367	0.182
Rotting flesh crawling with worms (2)	0.096	**0.644**	0.425	0.253
Skin infections/diseases	0.220	**0.555**	0.097	0.150
Decomposing animal carcass (3)	0.202	0.192	**0.542**	0.423
Stepping in dog feces (3)	0.394	0.273	**0.580**	0.202
Moldy food	0.370	0.286	**0.569**	0.094
Putrid or stagnant water	0.421	0.356	**0.529**	0.121
Rotting garbage	0.374	0.238	**0.674**	0.175
Rotting meat (3)	0.257	0.305	**0.518**	0.203
Liquid that comes out of the rubbish (3)	0.374	0.290	**0.651**	0.116
The smell of garbage	0.503	0.191	**0.683**	0.115
Sour milk	0.263	0.137	**0.644**	0.191
A dog eating feces	0.520^*^	0.115	**0.527**	0.172
Sewage	0.527^*^	0.337	**0.608**	0.101
Open animal carcass	0.089	0.240	**0.587**	0.517^*^
Decomposing human carcass	0.101	0.187	540^*^	**0.591**
Dead, disfigured body (4)	0.107	0.176	0.334	**0.724**
Tumors	0.306	0.220	0.159	**0.616**
Animal entrails	0.298	0.199	0.325	**0.570**
Exposed intestines (4)	−0.012	0.252	0.259	**0.788**
Exposed brains (4)	0.076	0.127	0.195	**0.750**
Human entrails	0.181	0.105	0.291	**0.737**
Gaping infected wounds oozing pus (4)	0.162	0.453	0.149	**0.557**
Open wounds	0.329	0.273	−0.089	**0.751**
Severe injuries (fractures, open wounds)	0.285	0.241	−0.231	**0.726**
**FACTOR LOADING TRENDS<0.512**				
Ingesting fecal matter (1)	**0.439**	0.155	0.397	0.310
A baby diaper (nappy) full of diarrhea	**0.398**	0.272	0.205	0.355
Kissing someone w/disgusting lips (e.g., smell/morphologic)	**0.474**	0.450	0.253	0.115
Crawling swarms of insects (2)	0.362	**0.472**	0.245	0.118
Severe acne (when there are big whiteheads, pus, etc.)	0.438	**0.496**	0.076	0.155
Dog shit	0.466	0.152	**0.476**	0.291
Eating of uncooked rotting masses (of any kind) (3)	0.188	0.334	**0.480**	0.192
Bugs, flies in food	0.306	0.401	**0.502**	0.150
Eating animal organs—brains, liver, tail, etc.	0.210	0.344	0.180	**0.363**
Vomit (4)	0.349	0.337	0.213	**0.387**

The upper section shows the items loading > 0.512 (bold) onto the corresponding 4 factors: Hygiene Issues, Parasite/Infection, Food/Environmental, and Injury/Viscera. The lower section shows the trends for items loading < 0.512 (bold). Items listed with bracketed numbers are those selected as representative of the numbered factor. Asterisks (

* *) denote items loading above 0.512 on more than one factor. These items were retained onto the factor of their highest loading*.

### Stage 5: factor analysis of pathogen items

#### Rating task objectives

The remaining 64 pathogen items were rated by another group of 111 participants (36 men, mean age ± *SD* = 36.9 ± 10.2, range 21–55; 75 women, age 35.7 ± 12.9, range 20–70) via an online survey. The survey was in English but country of origin was not collected. The survey asked participants two demographic questions—gender and age, and then to rate the 64 items, delivered in a randomized order for each participant, for disgust on a 7-point scale (0 = *not at all disgusting*, 6 = *extremely disgusting)*. These ratings provided a ranked order of the remaining items, as shown in Table 1.

#### Factor extraction

We conducted exploratory factor analysis in order to investigate underlying structure of the data and to aid in further item reduction. We based our choice of factor analysis method and rotation on two main assumptions, (1) the 64 items likely correlate to some degree on disgust in general, and (2) the analysis will result in distinct, easily interpretable, uncorrelated components of disgust. Based on recommendations for these assumptions (Field, 2013), we conducted a principal components analysis (PCA) with orthogonal rotation (Varimax with Kaiser normalization). The Kaiser-Meyer-Olkin measure of adequacy (KMO = 0.84) and Bartlett's test of sphericity (*p* < 0.001) both indicated a sufficient shared amount of common variance between the individual items to support this analysis. In order to determine which factors to extract from the data, two main criteria were used: (1) a visual scree plot (Cattell, 1966), to visualize the inflection in the slope along the mapped eigenvalues, and (2) a comparison between the initial eigenvalues >1 and the inflection shown in the scree plot. The scree plot showed that the inflection would justify retaining four factors. These four factors are also the only factors with eigenvalues >2. Twelve factors had eigenvalues >1, however, the first largest jump in eigenvalue rested between factors 4 (2.587) and 5 (1.964), thus justifying the extraction of four factors. These four factors cumulatively accounted for 58.91% of the variance.

For due diligence, two more tests to justify four-factor extraction were included. We re-ran the analysis using the four-factor extraction specification, which then provided post-extraction communality scores as well as the percentage of non-redundant residuals with absolute values >0.05. The overall average of the communalities was 0.59, and fit closely to Kaiser's recommended criterion for accuracy in determining the number of factors to extract (as cited in Field, 2013; and Stevens, 2002). Second, Field (2013) notes that the percentage of non-redundant residuals with absolute values >0.05 is indicative of how well the data fits the model, where the smaller the percentage (no more than 50%) the better the model fit. In this dataset, only 684 non-redundant residuals had absolute values >0.05 (33%), suggesting an acceptable model fit. Table 2 shows the loadings for these four factors after rotation. The items clustered into four components labeled as: Hygiene Issues (Factor 1), Parasite/Infection (Factor 2), Food/Environmental (Factor 3), and Injury/Viscera (Factor 4). Ten of the items failed to load above 0.512, the minimum loading value recommended by Stevens (2002) for sample sizes of 100. Four items cross-loaded onto more than one factor and were subsequently removed from further analyses: “a dog eating feces,” “sewage,” “open animal carcass,” and “decomposing human carcass.” The lower part of Table 2 is ordered the same way as the upper part but shows the loadings that fall below the threshold only, i.e., it illustrates the trend of the items' loadings onto the factors.

Cronbach's *alpha* (α = 0.978) indicated high internal consistency across the ratings of the 64 text items, and could not be increased by deleting any of the 64 items. Cronbach's *alpha* scores across each of the individual factors also indicated internal consistency for each factor (Factor 1: α = 957; Factor 2: α = 0.926; Factor 3: α = 0.938; and Factor 4: α = 0.947; α could not be increased in any of the factors by deleting any of the items within them). The four retained factors were then used in Stage 6 for the generation of the final image set.

### Stage 6: image-item selection and image generation

#### Decision rules for image-item selection

We chose to represent five items from each of the four factors, resulting in a total of 20 images. These items were selected by following a set of decision rules designed to reduce subjectivity in item selection, taking into account both the disgust ratings of the 64 items and their respective factor loadings. First, we focused on items that loaded above the threshold (0.512) in only one factor; we excluded from subsequent decisions any items that loaded above the threshold in more than one factor in order to draw a distinct boundary between factors. Within the remaining items loading onto each factor, we selected the four which had the highest overall disgust rating, according to the ranked order shown in Table 1. For example, of the items loading onto the “Hygiene Issues” factor, the one with the highest overall disgust rating is “dirty sanitary items” (loading = 0.634, mean rating = 3.75); therefore this item was selected. However, because some of the items in each factor are somewhat similar, we applied a third rule to avoid selection of similar items: only items that were considered to be distinct from the previous selected item(s) were selected. For example, based on the disgust rankings, the item “maggots in the wound of a living human” should be the third item selected from the “Parasite/Infection” factor. However, because it is more similar to the first two selections (“rotting flesh crawling with worms” and “worms in food…”) for this factor, we skipped this item, as well as “parasites that grow in humans,” but selected the next highest ranking item that loaded on this factor, “flesh-eating disease.” These rules were applied across the factors, generating 16 items. Finally, we further selected one item per factor from the factors' trend loadings (items loading below 0.512), because these items included the two highest-ranking disgust scores in Table 1 (“ingesting fecal matter” and “eating uncooked rotting masses”). Thus, within this group of items, we selected the item with the highest disgust score. This procedure resulted in the final total of 20 items to be depicted in the final image set, with 5 items from each of the 4 factors. The complete list is shown in Table 3.

**Table 3:** The 20 pathogen-salient items selected for depiction in final image set.

	**Hygiene Issues (F1)**	**Parasite/Infection (F2)**	**Food/Environmental (F3)**	**Injury/Viscera (F4)**
Item 1	Dirty sanitary items	Rotting flesh crawling with worms	Decomposing animal carcass	Dead, disfigured body
Item 2	Dirty/unflushed toilets	Worms in the food	Rotting meat	Gaping, infected wounds oozing pus
Item 3	Bad dental hygiene	Really dirty, fungus-infected toenails	Liquid that comes out of the rubbish	Exposed intestines
Item 4	When people eat their snot/bogeys	Flesh-eating disease	Stepping in dog feces	Exposed brains
Item 5	Ingesting fecal matter	Crawling swarm of insects	Eating uncooked rotting masses	Vomit

#### Generating the images

**Figure 1:**
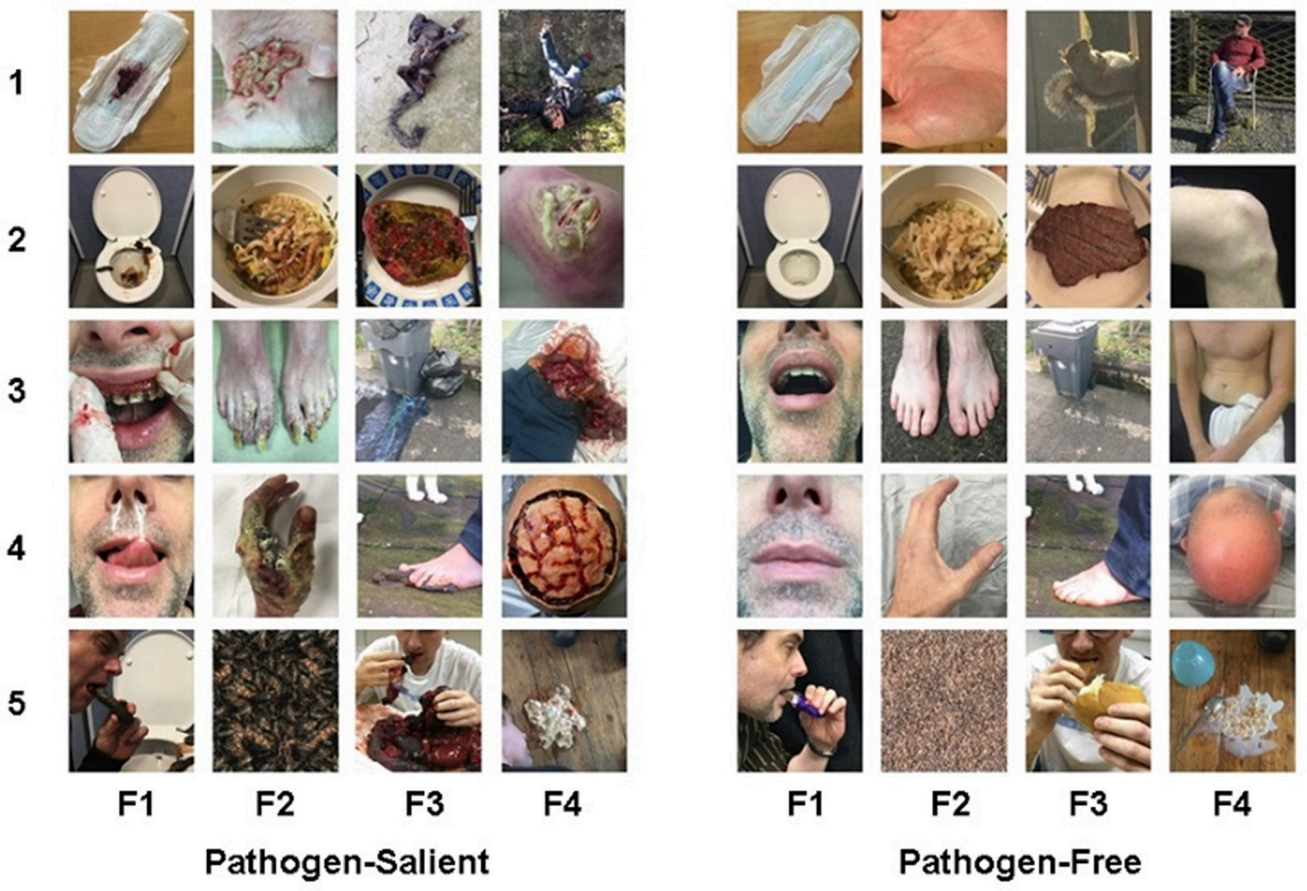
The Culpepper Disgust Image Set. Twenty pathogen-salient images (left) with their matching pathogen-free counterparts (right). F1–F4 represent the four disgust factors. F1, Hygiene Issues; F2, Parasite/Infection; F3, Food/Environmental; F4, Injury/Viscera (The orientation for images 1 and 4 in F2, and image 1 in F3 has been adjusted from landscape to portrait for the purpose of this collage).

Images were generated to represent, as closely as possible, the final 20 items. To gather some generalized ideas of what the public considers to be illustrative of the text of each item, we conducted an internet search (Google.com) using the exact item-wording of the individual items. Scenes were then prepared to closely, but uniquely, represent a generalized version of the collective group of item-images retrieved. We prepared the scenes for 19 of these images, in 8 of which we enlisted the help of professional special effects artists; for the remaining item (“decomposing animal carcass”), a photograph was taken of a real dead squirrel. Full color photographs were taken of each prepared scene. Furthermore, following Curtis et al. (2004), we also generated a matching image which lacked pathogen relevance but was otherwise similar. For example, for the disgust image depicting “dirty/unflushed toilet,” the matching image was of a clean/flushed toilet. We thus created 20 paired images−20 pathogen-salient images, each with a matching pathogen-free counterpart image (Figure 1). Each of the 40 images were created to provide as similar degree of focus, depth, and clarity as possible. They are uniformly sized—some images at 400 × 600 pixels in portrait and some at 600 × 400 pixels in landscape orientation (where both images of each individual image-pair are formatted in the same orientation). For use of C-DIS, see ESM.

### Stage 7: validation of the image set

#### Survey objectives

The aims of this final stage were two-fold. First, we aimed to compare differences in ratings between the pathogen-salient and pathogen-free images in the new image set, with the clear expectation that the pathogen salient images should elicit higher mean disgust scores than their pathogen-free counterparts; if so, then the new set (the C-DIS) can be considered effective as an instrument for eliciting disgust (by simply exposing people to the pathogen-salient images) or for measuring disgust sensitivity (comparing the difference between an individual's scores for the pathogen-salient and pathogen-free images). Second, we aimed to compare these scores with those elicited by the Curtis et al. (2004) image set. As noted earlier, to be considered an improvement over that set the C-DIS must elicit (1) a significantly larger overall mean disgust score for the pathogen-salient images compared to the pathogen-salient images in the Curtis set, (2) no significant increase (or some reduction) in the overall mean disgust score for the pathogen-free images compared to the disgust score for the pathogen-free image in the Curtis set, and therefore (3) a significantly larger difference in overall disgust ratio between the pathogen-salient and pathogen-free images compared to that of the Curtis set.

#### Participants and procedure

To meet these objectives, we constructed an online survey which included 54 images—the 40 new images (20 pathogen-free, 20 pathogen-salient) and Curtis' 14 images (7 pathogen-free, 7 pathogen salient). The images were resized to 350 × 500 and 500 × 350 pixels (corresponding to orientation) to better fit the survey pages. A link to the survey (on the Qualtrics.com platform) was shared through social media.

A total of 135 people responded to the survey link. Only participants over 18 years were recruited. For ethical reasons, the survey did not enforce responses to items and some participants did not provide ratings for every image; we therefore excluded eight participants who missed out more than two C-DIS pairs or one of the Curtis image pairs. The remaining 127 participants (mean age = 33.18 years, *SD* = 12.99, range = 18–66) included 46 men (36%), 79 women (62%), and 2 transgender (.01%). The native country for these participants were, in order of percentage: Colombia = 25 (20%), USA = 25 (20%), UK = 23 (18%), the Czech Republic = 17 (13%), Lebanon = 8 (6%), Germany = 7 (5%). Seventeen other countries were represented by 2 or less individuals, ordered alphabetically: Australia, Canada, Egypt, Ireland, Italy, Norway, Pakistan, Slovakia, Spain, Syria, Sweden, The Netherlands, and Turkey. Of these, participants' ethnic background included White (*n* = 103; 81%), Black/African descent (*n* = 4; 3%), and “Other” (*n* = 20; 16%), which included descriptions such as Native American/Alaskan, Asian, Latin American, Mestiza, Mexican, Middle Eastern, and Arab.

Participants were presented with the 54 images sequentially and in a fully randomized order that was unique to each participant. For each image, they were asked to rate it for disgust on a 7-point scale (0 = *not disgusting at all*, 6 = *extremely disgusting*).

#### Analyses

For each participant, we computed mean ratings for the pathogen-salient images and the pathogen-free images in each image set. Mean difference ratios were also calculated, by dividing the pathogen-salient image scores by the pathogen-free image scores for each image pair (a high ratio thus indicates that the pathogen-salient images were judged to be particularly disgusting compared to their controls). These same scores were also calculated for each of the four factors in the C-DIS: (1) Hygiene Issues, (2) Parasite/Infection, (3) Food/Environmental, and (4) Injury/Viscera.

The distribution of the data was explored for normality through visual inspection of the Normal Q-Q plot of the mean difference scores (pathogen-free subtracted from pathogen-salient mean scores) for each corresponding analysis rather than via Shapiro-Wilk test outputs, which are not recommended for sample-sizes >50 (Elliott and Woodward, 2007). The data met the assumptions of parametric tests.

Furthermore, in order to verify adequate statistical power, an *a priori* power and sample size analysis was performed using the Statistics Calculators Website (Soper, 2017) and the guidance of Cohen (1992). We calculated the anticipated effect size *d* = 0.80 at the statistical power level of.80, with a type I error rate of α = 0.01, and found that a minimum total sample size of *N* = 78 is required. Based on our sample size of *N* = 127, sufficient power to detect even a moderate difference was expected.

#### Internal consistency of image sets, intra-image sets, and factors

Internal consistency was assessed as an estimate of reliability by calculating Cronbach's *alpha* scores on each of the two image sets, on the pathogen-salient and pathogen-free sets within each full image set, and within each of the factors of the C-DIS.

##### C-DIS

The results indicate high internal consistency for the C-DIS as a whole (α = 0.946). Internal consistency was high for both the pathogen-salient (α = 0.944; item variance 0.527) and pathogen-free images (α = 0.932; item variance 0.177). There was also high internal consistency for individual factors in both the pathogen-salient set (Hygiene Issues, α = 0.810; Parasite/Infection, 0.787; Food/Environmental, 0.846; Injury/Viscera, 0.848) and the pathogen-free set (Hygiene Issues, α = 0.807; Parasite/Infection, 0.731; Food/Environmental, 0.712; Injury/Viscera, 0.765).

##### Curtis image set

The results indicate high internal consistency for the Curtis image set as a whole (α = 0.870), as well as for the pathogen-salient (α = 0.789; item variance 0.643) and pathogen-free sets (α = 0.766; item variance 1).

We also calculated Cronbach's *alpha* on the full C-DIS and Curtis sets individually to assess the internal consistency of each set within the four largest subsets of raters split by country of origin: Colombia, USA, UK, and the Czech Republic. There was high internal consistency for C-DIS within each country of origin: Colombia (α = 0.957), USA (α = 0.960), UK (α = 0.945), and the Czech Republic (α = 0.966). There was also high internal consistency for the Curtis set within each country of origin: Colombia (α = 0.896), USA (α = 0.874), UK (α = 0.884), and the Czech Republic (α = 0.922).

Overall, the individual images within each analyzed set showed similar degree of internal consistency to their corresponding set image cohorts. *Alpha* scores after item (image) deletion indicated that the internal consistency of each set could not be increased by removing any of the images within their corresponding set. Further, none of the images scored under α = 0.610, and the majority of the scores were above α = 0.750. The images, the intra sets, and the full image sets showed strong internal consistency as measured across a varied cross-cultural sample of individuals, which lends reliability, accuracy and, therefore, strength to the subsequent findings.

## Results

### Image-set correlations

We first correlated the mean scores for disgust given by participants to the C-DIS pathogen-salient images and the Curtis pathogen-salient images. A strong positive correlation was found between the two measures, Pearson *r*_(127)_ = 0.774, *p* < 0.001, showing that the C-DIS and Curtis sets affected raters similarly and suggesting that they measure responses along the same construct. We then proceeded to compare the image sets in more detail.

### Disgust ratings

Table 4 shows the mean disgust ratings for each of the pathogen-salient and pathogen-free images in the C-DIS and Curtis image sets. Among the C-DIS, pathogen-salient images were judged to be significantly more disgusting than their paired pathogen-free version (paired-samples *t-*tests, *p* < 0.001 in every case). Paired-samples *t*-tests were also conducted to compare the mean disgust scores between pathogen-salient and pathogen-free images representing each of the four factors (Table 5; here, grand means were calculated for each factor by averaging their 5 constituent item means). Again, grand means for pathogen-salient disgust ratings were significantly larger, for each factor, than the pathogen-free scores.

**Table 4:** Mean disgust scores for pathogen-salient and pathogen-free images, the difference ratio of how much more disgusting the salient images are compared to their pathogen-free counterparts, and the results from paired-sample *t*-tests for each image pair.

**Images**	**Pathogen**		**Difference ratio**	***t***	***p***
	**Salient**	**Free**			
**C-DIS**					
Dirty sanitary items	3.90	1.49	2.61	15.46	<0.001
Dirty/unflushed toilets	6.14	1.78	3.43	31.54	<0.001
Bad dental hygiene	5.79	1.68	3.43	28.69	<0.001
Eating snot/bogeys	5.23	1.63	3.19	24.04	<0.001
Ingesting fecal matter	6.22	1.43	4.34	34.41	<0.001
Rotting flesh crawling w/worms	6.31	1.32	4.76	40.70	<0.001
Worms in the food	5.20	1.75	2.96	20.72	<0.001
Dirty, fungus-infected toenails	6.17	1.93	3.18	29.82	<0.001
Flesh-eating disease	6.07	1.41	4.28	33.94	<0.001
Crawling swarm of insects	3.57	1.89	1.88	9.80	<0.001
Decomposing animal carcass	3.91	1.18	3.30	16.33	<0.001
Rotting meat	4.49	1.76	2.54	16.13	<0.001
Liquid coming out of rubbish	3.52	1.63	2.15	13.04	<0.001
Stepping in dog feces	5.25	1.74	3.02	23.35	<0.001
Eating uncooked rotting masses	5.16	1.45	3.54	22.41	<0.001
Dead, disfigured body	4.14	1.17	3.52	16.94	<0.001
Infected wound oozing pus	5.56	1.69	3.29	25.41	<0.001
Exposed intestines	5.26	1.48	3.53	24.47	<0.001
Exposed brains	4.10	1.53	2.67	14.48	<0.001
Vomit	5.05	1.64	3.07	23.93	<0.001
Overall mean	5.05	1.58	3.23		
**CURTIS IMAGE SET (ORIGINAL STUDY)**					
Plate of bodily fluid	3.14 (2.6)	1.71 (1.6)	1.83 (1.62)	10.70	<0.001
Person looking ill	2.25 (3.1)	1.38 (1.5)	1.62 (2.06)	8.26	<0.001
Crowded train carriage	1.70 (2.0)	1.38 (1.2)	1.22 (1.66)	3.78	<0.001
Towel stained/bodily secretions	3.75 (3.9)	1.55 (1.6)	2.41 (2.43)	15.03	<0.001
Skin lesion/pus-inflammation	5.34 (4.6)	3.07 (3.6)	1.73 (1.27)	16.76	<0.001
Gastro-intestinal worm	3.55 (3.8)	3.16 (3.7)	1.12 (1.02)	2.22	0.029
Louse	2.62 (3.5)	1.94 (2.8)	1.35 (1.25)	5.16	<0.001
Overall mean	3.19 (3.4)	2.03 (2.3)	1.61 (1.62)		

*Top, C-DIS; Bottom, Curtis image set, mean values from the original Curtis et al. (2004) study shown in brackets*.

**Table 5:** Mean disgust scores for pathogen-salient (PS) and pathogen-free (PF) images, the mean (and standard error) difference ratio (PS/PF), and results of *t*-tests comparing PS and PF sets, for each of the four factors identified by exploratory factor analysis of disgust items.

**Factors**	**Mean PS**	**Mean PF**	**Difference ratio**	***S.E*.**	***t***	***df***	***p***
1 Hygiene Issues	5.44	1.61	3.38	0.10	39.23	126	<0.001
2 Parasite/Infection	5.47	1.66	3.30	0.10	38.41	126	<0.001
3 Food/Environmental	4.47	1.55	2.88	0.11	25.69	126	<0.001
4 Injury/Viscera	4.83	1.50	3.22	0.12	27.62	126	<0.001

Table 4 also shows the equivalent scores for the Curtis images as provided by our raters, as well as (for purpose of comparison) the scores provided by the original raters in the Curtis et al. (2004) study. As would be expected, our raters awarded significantly higher disgust scores to pathogen-salient images than the pathogen-free control images, which provides justification for a more direct comparison of the two image sets using the ratings we collected.

### Comparing C-DIS and the curtis image set

#### Interaction and main effects

**Figure 2:**
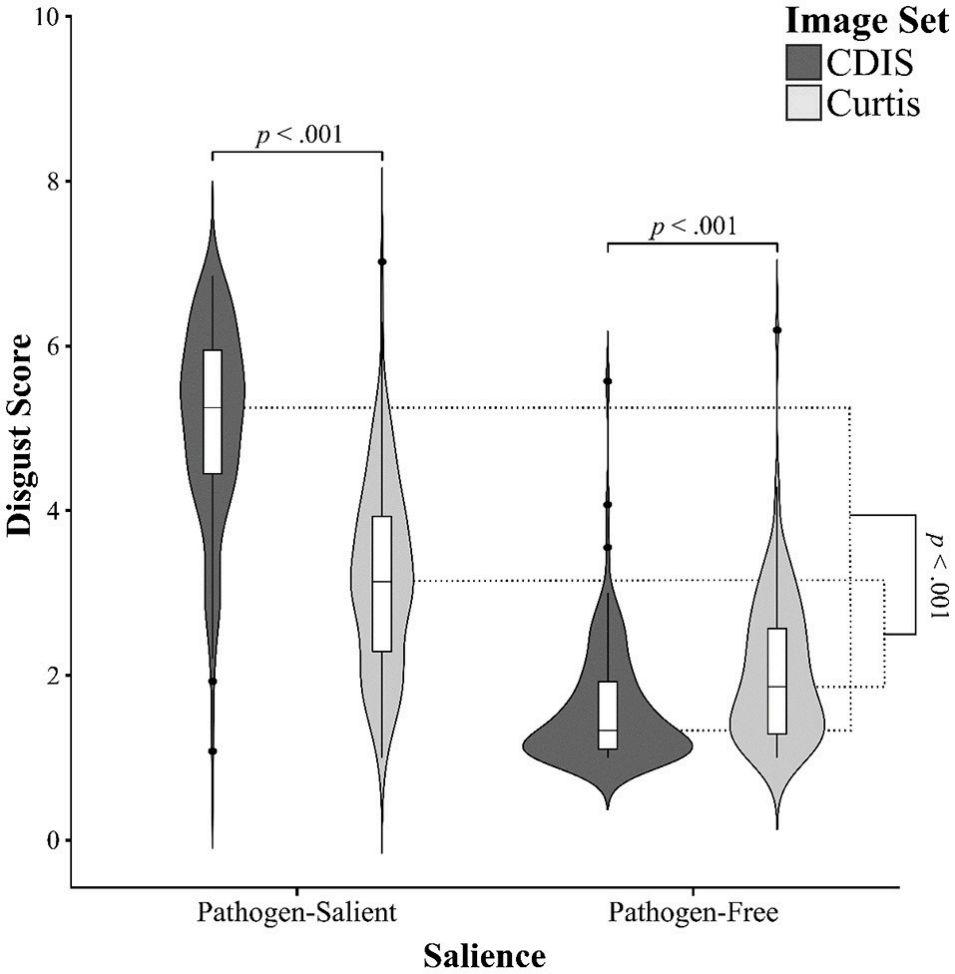
Kernel probability density (violin) plots with boxplots for disgust scores, split by image salience (pathogen-salient, pathogen-free) and image set (dark gray: C-DIS; light gray: Curtis). See text for statistical comparisons.

To compare the two image sets directly, we used a two-way repeated measures ANOVA with both image set (C-DIS, Curtis) and image type (pathogen-salient, pathogen-free) as within-subjects factors. In addition to the expected main effect of image type, with higher disgust scores for pathogen-salient images, *F*_(1, 126)_ = 1219.81, *p* < 0.001, η_p_^2^ = 0.906, there was a main effect of image set, *F*_(1, 126)_ = 344.25, *p* < 0.001, η_p_^2^ = 0.732, with higher disgust scores in the C-DIS, due to particularly high ratings in the pathogen-salient condition (Figure 2). More importantly, we found a significant interaction between image set and image-salience, *F*_(1, 126)_ = 667.46, *p* < 0.001, η_p_^2^ = 0.841. Pairwise *post-hoc* tests confirmed that the pathogen-salient disgust scores were significantly higher for the C-DIS than the Curtis set, *t*_(126)_ = 27.22, *d* = 2.5, while the pathogen-free versions were awarded lower disgust scores in the C-DIS compared with the Curtis set, *t*_(126)_ = 9.59, *d* = 0.45.

#### Difference ratios

Subsequently, we correlated difference ratios between pathogen-salient and pathogen-free scores of the C-DIS and the Curtis set. A significant, nearly moderate, positive correlation was found between the two measures, Pearson *r*_(127)_ = 0.283, *p* < 0.001, showing that disgust sensitivity of individual raters was affected and assessed in a similar way by each set.

As suggested by the significant image set × image type interaction, the mean difference ratio for the C-DIS images was significantly larger than for the Curtis set. On average, the pathogen-salient images in the C-DIS were judged by our raters to be 3.23 times more disgusting than the pathogen-free images (range = 1.88–4.76), compared with 1.61 (range = 1.12–2.41) times for the Curtis set (and 1.62 times as scored by the original raters in that study). With respect to individual image pairs, the difference ratios were larger for C-DIS image pairs than those for the Curtis images in every case except two: “crawling swarm of insects” and “liquid coming from the rubbish” (see Table 4). In addition, we observed that the overall mean difference ratios between pathogen-salient and pathogen-free images for each C-DIS factor (shown in Table 5) were larger than the Curtis set as a whole.

To obtain a direct comparison between image sets, we conducted a one-way ANOVA to compare the mean difference ratios for the individual image pairs across the C-DIS (*N* = 20), our current ratings of the Curtis set (*N* = 7) and those from the original study (*N* = 7). Mean difference ratios were significantly different between image sets, *F*_(2, 31)_ = 27.94, *p* < 0.001, η_p_^2^ = 0.643. Tukey *post-hoc* tests revealed that the mean difference ratio for C-DIS (3.23) was significantly larger than for both the current (1.61, 95% CI [0.94–2.29], *p* < 0.001) and original (1.62, 95% CI [0.95–2.29], *p* < 0.001) ratings of the Curtis set. Importantly, there was no difference between the ratios generated by our current ratings of the Curtis set and those in the original Curtis study (*p* = 0.999), reinforcing the earlier finding that our raters assessed those images in the same way and that other differences between the image sets cannot be attributed to unusual ratings in our study.

## Discussion

We have reported a 7-stage, bottom-up process culminating in a new image set (the Culpepper Disgust Image Set, C-DIS) which contains 20 pathogen-salient and 20 paired pathogen-free images. The multi-stage process was critical in order to generate a comprehensive overview of what people find disgusting, across different parts of the world, and how these triggers of disgust are inter-related. Importantly, the fact that the images were generated by the researchers, rather than being gleaned from the internet (for example), has two key advantages: it is possible to ensure that in every case the pathogen-free “control” images are appropriately matched to their pathogen-salient counterparts, and furthermore, from a practical point of view, the images are available to be used freely by researchers without copyright or ethical concerns.

We anticipate that the C-DIS can be used in two ways: (i) to activate pathogen disgust in participants in a treatment condition (i.e., through exposure to the pathogen-salient set) compared with a control group (i.e., participants who see the pathogen-free set), or (ii) as a tool to assess individual participants' pathogen disgust sensitivity (i.e., asking them to score both pathogen-salient and pathogen-free images and subsequently calculating difference scores).

### Effectiveness and improvement

There was a strong positive correlation between scores given by individual participants to the pathogen-salient images in both image sets. This indicates convergent validity in ability to elicit the emotion of disgust across the two image sets: if the Curtis pathogen-salient images are judged to trigger disgust, then the C-DIS pathogen-salient images appear to have a similar effect. Furthermore, the significant positive correlation between pathogen-salient: pathogen-free difference ratios in the two image sets also demonstrates convergent validity in the potential for assessing disgust sensitivity. In other words, individual participants who were especially (relative to other participants) disgusted by the Curtis pathogen-salient images compared with the pathogen-free images, and could therefore be said to have high disgust sensitivity, would also be found to have high disgust sensitivity based on responses to the C-DIS images.

Notwithstanding these between-set correlations, we conducted several analyses to determine the effectiveness of the C-DIS as a trigger of disgust and to compare its effectiveness against the images in the Curtis et al. (2004) image set. The analyses indicate that the bottom-up approach has resulted in an image set that is both effective as an experimental instrument and as an improvement to the Curtis set.

Considering the effectiveness of the C-DIS, the disgust scores for the pathogen-salient images were significantly larger than those for the pathogen-free images. The significant difference between these two scores suggests that the pathogen-salient images did elicit the desired effect—disgust—while the pathogen-free images served as effective “non-disgusting” controls to their salient-image counterparts. This is further supported by the difference ratio calculations between the C-DIS intra-sets representing each of the four identified underlying factors.

The comparisons between the two image sets indicate that each of our three improvement criteria were met. The mean disgust scores for our pathogen-salient images were significantly larger than for the pathogen-salient images in the Curtis set, suggesting that the C-DIS images activate the disgust response more strongly (*criterion 1*). Furthermore, the mean disgust scores for our pathogen-free images were significantly lower than the pathogen-free images from the Curtis set (*criterion 2*). This reduces the chance, in future experimental studies, for disgust to be unintentionally elicited in participants in the control condition. As a result of these properties, the difference ratios between pathogen-salient and pathogen-free images for the C-DIS were significantly larger than the difference ratios in the Curtis set (*criterion 3*). Larger difference ratios increase the efficacy of any manipulation of disgust, if either pathogen-salient or pathogen-free images are seen by treatment and control groups, respectively. They should also increase the ability to discriminate between different levels of disgust sensitivity, if individual participants are asked to judge both kinds of image.

There are two further advantages worth noting. One relates to the number of images: 20 pathogen-salient (and matched pathogen-free) images in the C-DIS set, whereas the Curtis set consists of only 7. In addition to the average potency of each individual image in eliciting disgust, the C-DIS should therefore also ensure a comparatively prolonged exposure to a more diverse set of pathogen threats when shown to participants in future research, providing an increased likelihood of more effectively activating the behavioral immune system. A second is the underlying structure of the C-DIS, differentiating between four different factors that contribute to pathogen-disgust. Our analyses showed that the disgust scores for pathogen-salient images were significantly larger than for the pathogen-free images in all four factors. This suggests that the four factors are, for the most part, equally supportive of the image set as a whole. As illustrated in Table 5, the mean pathogen-free scores for each individual factor in the C-DIS is smaller than the overall mean pathogen-free score for the Curtis set (whether the latter is determined using raters in our study or those in the original Curtis et al. study; see Table 4). Similarly, the overall mean pathogen-salient score is larger for each C-DIS factor than both Curtis measurements (current and original), as are the overall mean difference ratios.

### The C-DIS and the DIRTI

The coincidental timing of the development of both the C-DIS and the DIRTI (Haberkamp et al., 2017) demonstrates recognition of the need for high-quality and validated image sets for the study of disgust. Both instruments importantly address methodological issues of the previously developed image sets, specifically target the disgust emotion, and elicit disgust along multiple factors. However, as the two image sets use different perspectives and have different aims, they thus have advantages that correspond to each individual approach. The C-DIS was designed from an evolutionary perspective to investigate the effects of BIS activation on human behavior, whereas the DIRTI was designed from a clinical perspective to be used for therapeutic and experimental purposes involving psychiatric disorders. There is considerable overlap from both perspectives in that psychiatric disorders such as phobias are considered to have evolutionary origins (Marks and Nesse, 1994; Öhman and Mineka, 2001; Nesse, 2005); therefore, the two sets do not discount each other, and in fact are likely to be complementary. However, compared with the six DIRTI categories (food, animals, body products, injuries/infection, death, hygiene), our analytical categorization suggests four underlying components to pathogen disgust and items are assigned to categories based on functional considerations rather than the clinical approach focusing on phobias. Thus, for example, the C-DIS treats injuries and infection as two distinct triggers of disgust, while the DIRTI combines them.

### Limitations

Despite the above, we acknowledge several limitations of this study. The first limitation concerns the nature of the sample. Participants for each stage of the study were recruited via online surveys distributed throughout various social media outlets and through universities. For example, in Stage 1 there were 460 participants of various ages and gender. While the study was cross-cultural to the extent that we solicited items that trigger disgust from participants across four countries, and had the final images similarly rated, our method of recruitment suggests that most of the participants were of reasonably WEIRD backgrounds (Westernized, Educated, Industrialized, Rich, and Democratic: see Henrich et al., 2010). For example, each participant had to have access to a computer with global internet service; they had to be somewhat educated in order to use a computer and read somewhat complex instructions; and they had to have, or at least have access to (e.g., via parents) the financial means that allow for such access to computers and education. Many of the participants were students or staff recruited at universities. This may have biased the kinds of items suggested by the participants in Stage 1. Future studies, including attempts to devise a new image set or to improve upon the set of images devised here, would do well to include non-WEIRD participants from an even wider geographical spread, if possible.

Demographic data was not collected regarding participant work experience or education, or of the topic of study by students and staff for any of the stages. This may have biased results in that, for example, participants working or studying in the medical field may be exposed to these types of disgust items more regularly than others. Repeated exposure to disgust items may reduce disgust sensitivity, which would have affected the overall disgust scores. Level of hunger was not recorded either, which could be important when attempting to measure disgust sensitivity. Hunger can induce disgust suppression for pathogenic foods (Al-Shawaf et al., 2014), which can have an effect on the disgust ratings for the pathogenic food images in future studies. Furthermore, as with all online studies, it is impossible to control whether participants are under the influence of stimulants (e.g., coffee, cigarettes) and other intoxicants or medication (e.g., alcohol, anti-anxiety) that can affect perception and dull senses (noted in Culpepper, 2014). For accuracy and validity, further studies should consider this variation in rater experience. Having said this, such issues should not have affected the specific comparisons we made between image sets. Furthermore, we used two slightly different rating scales in the stages of the study that involved ratings. Although within each stage we were interested in the relative disgust ratings rather than absolute scores, use of the same scale throughout the procedure would be useful in future work.

In Stage 4, it was necessary to make some judgments regarding item distinctiveness. For example, we conflated the individual items “cat vomit,” “children's vomit,” and “vomit” into an umbrella category “vomit.” It is possible that some of the items lost in this process might have been rated more disgusting than the resulting umbrella term. However, the decision could be justified in that it was likely to be conservative in effect, and it is unlikely that the basic items would be visually distinguished from images in any case. It is therefore unlikely that these rare unification instances jeopardized the integrity of the process.

Finally, although the results of Stage 6 and 7 suggest that the images accomplished the goal they were devised to accomplish, the decisions of how to depict these items and scenarios in their respective images were somewhat subjective. However, we attempted to reduce this through the initial internet search on specific item wordings, selecting a scene that best represented the images generated by the search.

## Conclusion

Overall, the current study set out to create a new set of disgust images that can be used in future experimental work on the behavioral immune system. We employed a bottom-up approach to devise a larger, more comprehensive, and arguably more representative set of images, constructed of items, scenes, and scenarios that trigger pathogen disgust, which is thought to be the most evolutionarily ancient domain of emotion (Schaller, 2006; Schaller and Duncan, 2007; Tybur et al., 2009). This is particularly important when considering research into this adaptation at a cross-cultural level. This methodological process resulted in a set of 20 cross-culturally determined and validated disgust images specifically designed to trigger pathogen disgust and activate the behavioral immune system. One of the main validation steps for this new set was to compare it against a set already available in the literature and used by other researchers, the Curtis et al. (2004) image set. The new set needed to (i) elicit pathogen disgust; to do so reliably (ii) in individuals, and (iii) in cross-cultural samples; (iv) to elicit disgust more strongly than Curtis' image set; and (v) exhibit larger differences between the pathogen-salient and pathogen-free sets compared to Curtis' set. Our results showed clearly that this cross-cultural, multi-staged, bottom-up process has produced a new set of disgust images that meet these requirements. We suggest that our image set is an effective instrument for consistently and reliably eliciting pathogen disgust and measuring pathogen disgust sensitivity across cultures. Moreover, it does so along four distinct pathogen disgust factors—something not previously done.

## Data availability

The data (electronic supplementary material, ESM) associated with this research are available at http://hdl.handle.net/11667/121.

## Author contributions

PC was responsible for design, data collection and analysis, and he was primary author of the manuscript. SR contributed to design, analysis, and manuscript. JH aided with data collection from Czech Republic, and contributed input on analysis and manuscript. JL aided with data collection from Colombia, and contributed input on analysis and manuscript.

### Conflict of interest statement

The authors declare that the research was conducted in the absence of any commercial or financial relationships that could be construed as a potential conflict of interest.
